# Periodontal therapy among patients with cardiovascular disease: exploring its association with lower medical spending

**DOI:** 10.3389/fpubh.2026.1741942

**Published:** 2026-03-17

**Authors:** Morgan Santoro, Caroline McLeod, Lisa J. Heaton, Hannah J. Cheung, John J. O’Malley, Madison Vinson, Rebecca A. Preston, Eric P. Tranby

**Affiliations:** CareQuest Institute for Oral Health, Boston, MA, United States

**Keywords:** cardiovascular disease, commercial insurance, gamma regression analysis, periodontal disease, propensity score matching

## Abstract

**Introduction:**

Cardiovascular disease (CVD) and periodontal disease are prevalent chronic conditions that share common risk factors and may be linked through systemic inflammation. Emerging evidence suggests that individuals with periodontal disease are more likely to be diagnosed with CVD, potentially influencing health care costs.

**Methods:**

This study analyzed deidentified commercial insurance claims data from 192,500 United States (U. S.) adults aged 21–64 with CVD diagnoses between 2015 and 2022. Patients were categorized based on periodontal treatment received in 2020–2021: none, 1–3 visits, or 4 + visits. Gamma regression and multinomial propensity score matching (PSM) models assessed associations between periodontal treatment and 2022 health care costs (overall, outpatient, inpatient, and prescription), controlling for demographic and clinical covariates. Reported percentage reductions and average treatment effect on treated (ATET) dollar difference reflect estimates derived from PSM-weighted models.

**Results:**

Patients with four or more periodontal visits had significantly lower overall medical costs (9.6% reduction; ATET = −$1,960.39, 95% confidence interval (CI) = −$2,684.33, −$1,236.44, *p* < 0.005) and outpatient costs (5.3% reduction; ATET = −$599.09, 95% CI = −$1,019.34, −$178.84, *p* < 0.005) compared to those without treatment. Patients with 1–3 visits had an 8% reduction in prescription costs (ATET = −$387.53, 95% CI = −$653.31, −$119.75, *p* < 0.005). Gamma regression analyses confirmed these associations after adjusting for confounders.

**Conclusion:**

Findings suggest that periodontal treatment may be associated with lower health care costs among individuals with CVD, potentially attributed to reduced systemic inflammation. The cost differences were most pronounced with four or more periodontal visits, supporting risk-based recall strategies. These results underscore the relevance of integrating oral health into chronic disease management and indicate that greater access to periodontal care may be associated with economic and health benefits.

## Introduction

Cardiovascular diseases (CVDs) are a group of conditions with two categories, diseases of the heart *and* diseases of the blood vessels that supply blood to different parts of the human body (1). Heart diseases include but are not limited to coronary heart disease, rheumatic heart disease, congenital heart disease (2, 3), arrhythmia, pericardial disease, and heart failure while blood vessel diseases include hypertension, cerebrovascular disease, peripheral artery disease, deep vein thrombosis/pulmonary embolism, and aortic disease. These conditions prevent adequate blood flow to the heart and brain and can subsequently lead to myocardial infarction (heart attack) and cerebrovascular accident (stroke).

In the United States (U. S.) nearly half (48.6%) of adults had some form of CVD between 2017 and 2020 (4) and it remains the leading cause of death (5). From 2021 to 2023, CVD was more common in older adults (ages 60+) than younger adults (ages 20–59) (3). Persons identifying as non-Hispanic Black were also more likely to report having CVD (61%) compared to those identifying as non-Hispanic white (48%), Hispanic (46%), or non-Hispanic Asian (44%) (3). Addressing certain risk factors like smoking, physical inactivity, poor nutrition/diet, poor sleep, excessive alcohol use, being overweight or obese, high cholesterol, diabetes, and high blood pressure can lower risks for developing CVDs and support management of the disease (4).

Like CVDs, periodontal disease is also a group of conditions that affect the periodontal tissues (gums and bone) that support the teeth (6). Gingivitis is inflammation limited to the gums and if left untreated, can progress into periodontitis which is characterized by destruction of bone surrounding the teeth and often tooth loss due to chronic inflammation (6). The inflammation seen in periodontal disease is caused by an imbalance (or dysbiosis) where harmful, or pathogenic, bacteria such as *Porphyromonas gingivalis*, *Aggregatibacter* *actinomycetemcomitans*, *Tannerella forsythia*, *Eikenella corrodens*, and *Fusobacterium nucleatum* overtake beneficial bacteria in plaque biofilm, driving an inflammatory response by the body (7). This inflammatory response not only affects the mouth but can affect other parts of the body, such as the cardiovascular system (8).

In the most recent prevalence estimate of periodontal disease collected between 2009 and 2014 (9), 42.2% of adults 30 years or older had periodontitis, of which 34.4% was non-severe and 7.8% was severe (10). Periodontitis was most prevalent among men (50.2%), adults living below 100% of the federal poverty level (60.4%), current smokers (62.4%), and individuals who self-reported having diabetes (59.9%) (10). Additionally, periodontitis was most prevalent among adults identifying as Hispanic (63.5%) and non-Hispanic Black (59.1%), followed by non-Hispanic Asian (50%); periodontitis was least common among non-Hispanic white adults (40.8%) (11). Addressing certain risk factors, some of which are shared with CVD, like smoking, poor nutrition/diet, poor oral hygiene, stress and teeth grinding, being overweight or obese, diabetes, and autoimmune diseases can lower risks for developing periodontal disease and support management of the disease (12).

General understanding of the links between CVD and periodontal disease continue to develop. Pathogenic periodontal bacteria have been found in atherosclerotic plaque and arterial walls (13) and linked to subclinical atherosclerosis (14), coronary heart disease (15), coronary artery disease (16), peripheral artery disease (17), cerebrovascular disease (17), atrial fibrillation / atrial flutter (17), stroke (17), myocardial infarction (17), and hypertension (17). Studies have shown that adults with periodontitis face an increased risk of stroke (17) and ischemic heart disease, with the risk of stroke notably higher compared to individuals without the periodontitis (18, 19). These links likely result from pathogenic periodontal bacteria directly invading cardiovascular tissues or creating inflammatory pathways that indirectly affect the cardiovascular system (20). Additionally, those with severe periodontitis were more likely to have history of coronary artery disease and stroke than those with mild or moderate periodontitis (21). One prospective study found that adults were at greater risk of developing peripheral artery disease if they had a periodontal disease diagnosis, prior periodontal treatment, or self-reported tooth loss due to periodontal disease (22).

While recent Cochrane systematic reviews provide no conclusive evidence of periodontal treatment on preventing CVD (23, 24), studies show links between reduced coronary heart disease inflammatory biomarkers with non-surgical periodontal treatment (25) and potential lower risk for CVD with toothbrushing twice a day for at least 2 min (26). Additionally, other evidence suggests that using statin drugs to treat atherosclerotic cardiovascular disease therapeutically treated periodontal disease through their anti-inflammatory mechanisms (27). Finally, recent updates from the American Heart Association synthesize the clear contributions of periodontal disease to the chronic inflammation of atherosclerotic cardiovascular disease, and its treatment may help prevent and manage CVD (20).

CVD and periodontal disease place a significant financial strain on the healthcare system. By 2035, it is estimated that CVD will cost the U. S. $1.1 trillion in direct costs (e.g., cost of medical services) and indirect costs (e.g., lost work and productivity). This is double the 2016 cost of $555 billion (28). The highest total CVD costs (direct and indirect) are expected among individuals aged 80 and older, surpassing costs associated with Alzheimer’s disease or diabetes (28). Working-age individuals (45–64) face the highest indirect costs from lost earnings and household productivity from premature death due to CVD (28). It is estimated that in 2018 alone, periodontal disease cost the U. S. health care system $3.49 billion in direct (periodontal care) and indirect costs (productivity loss, edentulism, dental caries (cavities)) (29).

Existing evidence, though mixed, suggests that treating periodontal disease may help reduce medical costs and healthcare utilization for individuals with CVD. One study found that patients with cerebrovascular disease and diabetes experienced 20–40% reductions in medical costs (inpatient and outpatient) and annual hospitalizations for up to 3 years following periodontal therapy compared to a control (30). Another study showed a decrease in inpatient costs but an increase in outpatient costs for coronary heart disease after periodontal treatment (31). A third study suggests that utilizing a dental insurance benefit to support adherence with preventive dental care (including periodontal care) was associated with significant average yearly cost savings for patients with coronary artery disease (32). The aim of our study was to examine the relationship between health care costs (overall, outpatient, inpatient, and prescription) and periodontal treatment. We hypothesized that adults age 21–64 with CVD who had at least one periodontal treatment will have lower health care related costs than adults age 21–64 with CVD who did not have periodontal treatment.

## Methods

### Study population

Data used in this study consisted of Merative deidentified commercial insurance administrative claims data from 2015 through 2022. The data set contained integrated enrollment, outpatient and inpatient medical, prescription, and dental claims information from select commercial insurance plans in all 50 states. Eligible claims for analysis were from adults aged 21 through 64 years who were continuously enrolled from 2018 to 2022.

Adults were included if they received a CVD-related diagnosis from 2015 through 2020 and continued to have CVD in 2021 and 2022. A CVD-related diagnosis during that period was determined by the presence of an ICD-10 code within the I00-I99 range (Diseases of the Circulatory System; see subtypes in Appendix A) (33, 34). The WCG IRB reviewed and approved this study (ANP0008, May 2018).

### Treatment and control groups

**Figure 1:**
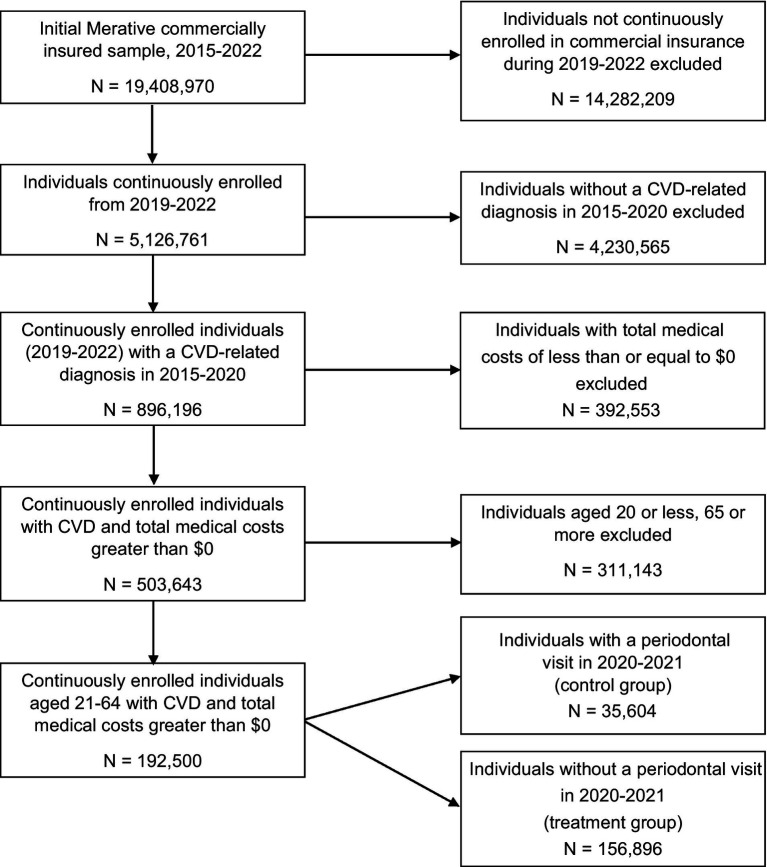
Flow diagram describing definition of final analytic sample.

Patients were grouped into treatment and control groups based on dental claims data showing use of periodontal services from 2020 to 2021 (Figure 1). Periodontal treatment was defined using *CDT 2024: Current Dental Terminology* codes D4000 through D4999 (see Appendix B) (35). Periodontal treatment was coded as yes if an adult with a CVD-related diagnosis had at least one periodontal treatment visit from 2020 through 2021 (treatment group) and was coded as no if an adult with a CVD-related diagnosis did not have at least one periodontal treatment visit from 2020 through 2021 (control group). A periodontal treatment visit was defined as any unique date of service where one or more periodontal CDT codes were submitted per enrollee. We examined the impact of both number of periodontal visits (none, 1–3, 4 or more) and type of periodontal treatment (surgical, non-surgical, or both), to account for severity of periodontal disease, on health care costs.

### Outcomes

The primary outcome measures of this study are overall medical costs, outpatient medical costs, inpatient medical costs, and outpatient prescription drug costs in 2022. These cost variables are the total amount paid to the provider through insurance and out-of-pocket costs. Overall medical costs represent the sum of outpatient and inpatient medical costs as well as outpatient prescription drug costs. This study examines the associations of periodontal treatment with these cost-related outcomes.

### Confounding variables

This study accounted for multiple confounding variables in the statistical model. These confounding variables included age (21 through 64 years) as a continuous variable; sex (male or female); metropolitan status (metro versus non-metro residence); and Elixhauser comorbidity score (no comorbidities, 1, 2, 3 or more) (36). Metropolitan status is derived from patients’ 5-digit ZIP codes mapped to the Metropolitan Statistical Areas (MSA) and subsequently collapsed into a binary indicator.

### Statistical methods

Descriptive statistics were used to summarize the use of periodontal services by patients with a CVD-related diagnosis from 2020 through 2021 as a proportion for number of periodontal visits (none, 1–3, 4 or more). We then summarized each of the outcomes and confounding variables according to use of periodontal services during 2020–2021 and insurance type. Two separate statistical models were run to examine the association between periodontal treatment in 2020–2021 and health care costs in 2022. Adults with CVD were divided by time recency of onset from the time from when costs were assessed in 2022.

Gamma regressions, a type of generalized linear model (GLM), were used to assess costs among utilizers for CVD patients with and without periodontal visits while controlling for age, sex, Elixhauser category, and metropolitan residence. For inpatient, outpatient, and prescription costs, zero costs reflect non-use of these health care services rather than “low cost” (e.g., the minimal cost of a single prescription or outpatient health care visit) and were therefore removed. Gamma regressions were chosen due to the right-skewed and strictly positive nature of the outcome variables. Gamma regressions estimate the mean of the skewed distribution, account for the non-constant variance, uses the link function to linearize the relationship, and fits the models using maximum likelihood estimation.

Propensity score matching (PSM) matches each patient in the treatment group to patient in the control group based on their propensity score. In other words, for each patient, the propensity score can be considered as the probability of receiving treatment (in this study, this is defined as having at least one or more periodontal visits). These PSM models predict total medical costs, total outpatient costs, total inpatient costs, and total prescription costs for all patients in the study, regardless of treatment or control group. All models controlled for age, sex, Elixhauser category, and metropolitan residence.

The periodontal visits variable was also broken out by no visits, one to three visits and four or more visits in 2020–21. Multinomial PSMs were run due to the treatment variable having more than two levels. In these models, weighting is conducted to adjust for confounding across the multiple groups, estimate the average treatment effects (ATE), and handle unequal group sizes and probabilities. Each patient receives a weight based on the inverse of the probability of receiving the treatment they did receive. These models looked at all CVD patients predicting total medical costs, total outpatient costs, total inpatient costs, and total prescription costs. All models controlled for age, sex, Elixhauser category, and metropolitan residence.

## Results

Table 1 presents the overall demographic characteristics of the sample as well as demographic characteristics stratified by number of periodontal treatments received (none, one to three, and four or more). Overall, 192,500 patients were identified as having at least one CVD diagnosis (mean age = 54.0 years [standard deviation (sd) = 8.0]; 45.5% female); the most commonly reported type of CVD was “other” (27%), followed by arterial CVD (7.7%) and hypertensive CVD (6.2%). Most (81.5%) patients had no periodontal treatment; 10% had one to three treatments, and 8.5% had four or more. Most patients lived in non-metropolitan areas (88%), and 60% had a score of four or higher on the Elixhauser comorbidity scale.

**Table 1:** Demographic characteristics of commercially-insured patients with CVD (*n* = 192,500), stratified by past use of periodontal services.

	Overall	Periodontal treatment		
		No periodontal treatment	1–3 periodontal visits	4 + periodontal visits
Total (*N*, %)	192,500	156,896 (81.5)	19,233 (10)	16,371 (8.5)
Age in years (mean, (SD))	54.0 (8.0)	54.0 (8.0)	54.0 (8.0)	56.0 (7.0)
Gender (*N*, %)				
Female	87,645 (45.5)	72,960 (46.5)	8,361 (43.5)	6,324 (38.6)
Male	104,855 (54.5)	83,936 (53.5)	10,872 (56.5)	10,047 (61.4)
Metropolitan residence				
Metropolitan	23,471 (12.2)	20,935 (13.3)	1,403 (7.3)	1,133 (6.9)
Non-metropolitan	169,029 (87.8)	135,961 (86.7)	17,830 (92.7)	15,238 (93.1)
Elixhauser comorbidity score				
0–1	20,869 (10.8)	17,534 (11.2)	1,767 (9.2)	1,568 (9.6)
2–3	56,086 (29.1)	45,863 (29.2)	5,300 (27.6)	4,923 (30.1)
4 or more	115,545 (60.0)	93,499 (59.6)	12,166 (63.2)	9,880 (60.4)
CVD diagnosis type (N, %)				
Hypertensive	11,993 (6.2)	9,529 (6.1)	1,347 (7.0)	1,117 (6.8)
Ischemic	21,769 (11)	17,487 (11)	2,212 (12)	2,070 (13)
Pulmonary	3,128 (1.6)	2,585 (1.6)	301 (1.6)	242 (1.5)
Other heart	51,721 (27)	43,116 (27)	4,740 (25)	3,865 (24)
Cerebrovascular	8,788 (4.6)	7,091 (4.5)	911 (4.7)	786 (4.8)
Arterial	14,753 (7.7)	11,952 (7.6)	1,503 (7.8)	1,298 (7.9)
Venous	1,811 (0.9)	1,519 (1.0)	161 (0.8)	131 (0.8)

SD, Standard deviation.

**Figure 2:**
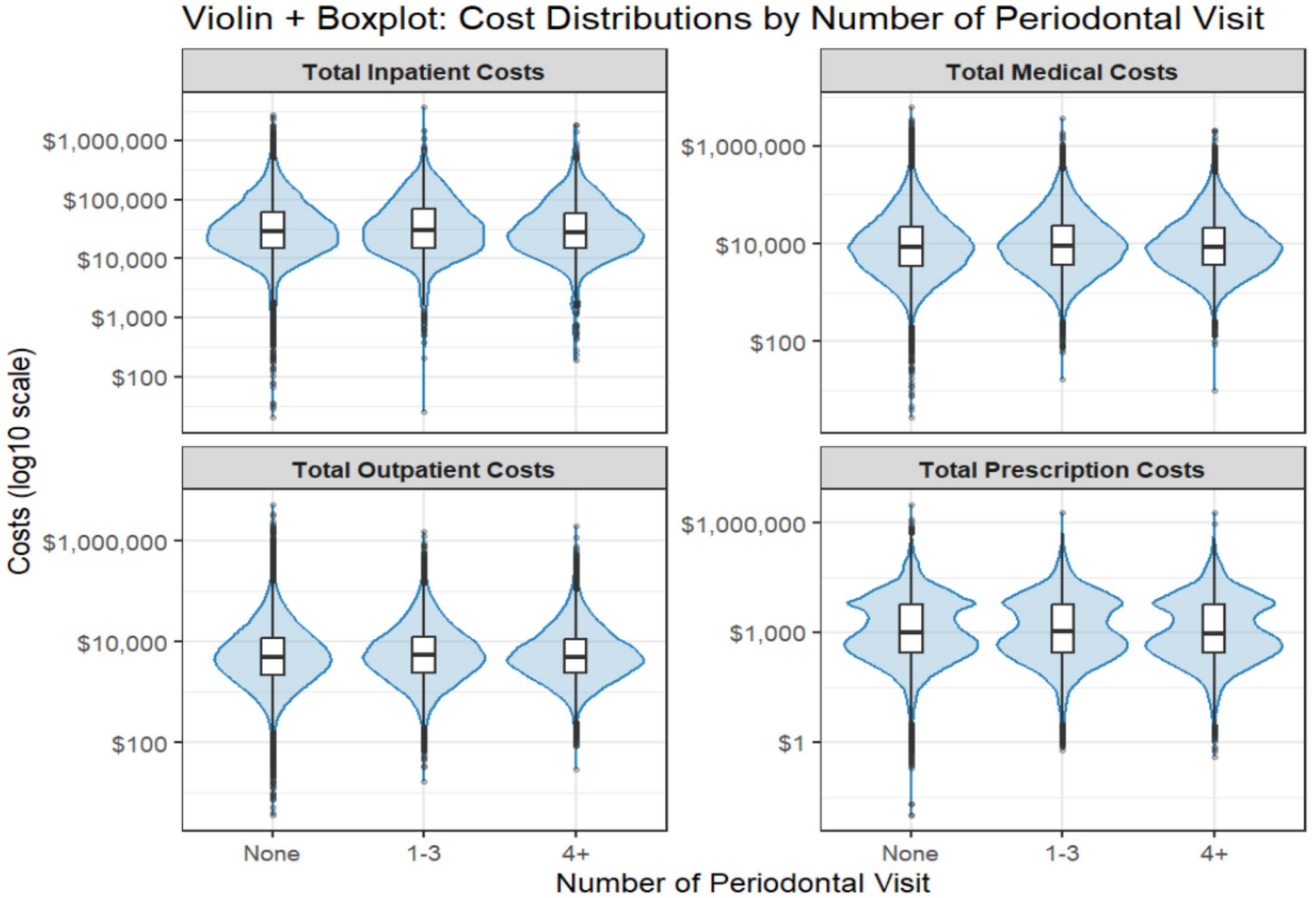
Cost distributions of overall medical, outpatient, inpatient, and prescription costs by past use of periodontal services. Error bars, Standard deviation.

Figure 2 displays the cost distributions related to overall medical care, outpatient care, inpatient care, and prescriptions by number of periodontal treatments received (none, 1–3, 4 or more); Table 2 shows the mean (sd) costs (overall, outpatient, inpatient, prescription) by type of periodontal treatments (none; non-surgical only; surgical only; both non-surgical and surgical). Patients who received one to three periodontal treatments were observed to have higher costs across three of four cost measures, compared to both those who received no treatment and those who received four or more treatments: overall health care costs [mean = $26,147 (sd = $65,597)], outpatient costs [mean = $13,836 (sd = $35,156)], and inpatient costs [mean = $6,224 (sd = $42,483)]. Only prescription costs were lower for individuals with one to three periodontal visits compared to other individuals [mean = $6,077 (sd = $22,843)]. Individuals with only surgical periodontal treatment had higher costs for overall medical care [mean = $26,362 (sd = $54,074)] and outpatient care [mean = $15,221 (sd = $33,655)] compared to individuals without periodontal treatment, those with non-surgical periodontal treatment only, and those with a mix of non-surgical and surgical treatment. Patients with no periodontal visits had higher inpatient costs [mean = $5,976 (sd = $37,809)] and prescription costs [mean = $6,332 (sd = $22,413)] than those with non-surgical, surgical, or mixed periodontal treatments.

**Table 2:** Mean (standard deviation) overall medical, inpatient, outpatient, and prescription costs for patients with CVD by type of periodontal treatment visits.

Standard deviation	Overall *N* = 192,500	No periodontal visits *n* = 156,922	Non-surgical only *n* = 30,962	Surgical only *n* = 2,576	Non-surgical and surgical *N* = 2,040	*p*-value
Overall medical costs						<0.001
Mean (SD)	25,784 *(67,232)*	25,966 *(68,003)*	24,933 *(65,030)*	26,362 *(54,074)*	23,894 *(54,044)*	
Median (Q1, Q3)	8,658 (3,490, 22,513)	8,637 (3,434, 22,615)	8,715 (3,637, 21,947)	9,316 (4,345, 23,564)	8,446 (4,065, 21,405)	
Outpatient costs						<0.001
Mean (SD)	13,589 *(41,052)*	13,658 *(42,357)*	13,116 *(34,753)*	15,221 *(33,655)*	13,364 *(35,417)*	
Median (Q1, Q3)	4,996 (2,191, 12,029)	4,946 (2,136, 12,052)	5,072 (2,375, 11,756)	6,449 (3,127, 13,838)	5,588 (2,864, 12,193)	
Inpatient costs						<0.001
Mean (SD)	5,897 *(38,102)*	5,976 *(37,809)*	5,627 *(40,810)*	5,000 *(27,712)*	5,067 *(27,951)*	
Median (Q1, Q3)	0.00 (0.00, 0.00)	0.00 (0.00, 0.00)	0.00 (0.00, 0.00)	0.00 (0.00, 0.00)	0.00 (0.00, 0.00)	
Prescription costs						<0.001
Mean (SD)	6,297 *(22,624)*	6,332 *(22,413)*	6,189 *(24,156)*	6,140 *(20,142)*	5,462 *(17,050)*	
Median (Q1, Q3)	860 (225, 5,311)	868 (227, 5,312)	836 (219, 5,400)	794 (207, 4,849)	764 (200, 4,600)	

SD, Standard deviation; all figures in US dollars.

Gamma regressions were used to assess all four types of health care costs for CVD patients with and without periodontal visits while controlling for confounding variables (Table 3). When controlling for age, gender, metropolitan residential status, and Elixhauser comorbidities, individuals who had four or more periodontal visits had significantly lower overall medical costs (*β* = 0.92, 95% confidence interval (CI) = 0.88, 0.95, *p* < 0.001) and outpatient costs (*β* = 0.94, 95% CI = 0.90, 0.99, *p* < 0.001) compared to those without periodontal treatment. Those with one to three periodontal visits had significantly lower prescription costs (*β* = 0.94, 95% CI = 0.89, 0.99, *p* < 0.05) than individuals without periodontal treatment.

**Table 3:** Gamma regression analysis results predicting average overall medical, outpatient, inpatient, and prescription costs for patients with CVD who received periodontal treatment [exp (β), (95% confidence interval)].

	Overall medical costs	Outpatient costs	Inpatient costs	Prescription costs
Number of periodontal visits				
None	ref	ref	ref	ref
1–3 visits	0.98 (0.95, 1.02)	1.00 (0.96, 1.04)	1.03 (0.95, 1.12)	0.94 (0.89, 0.99)*
4 or more visits	0.92 (0.88, 0.95)**	0.94 (0.90, 0.99)*	0.97 (0.88, 1.07)	0.96 (0.91, 1.02)
Age	1.00 (0.99, 1.00)**	1.0 (0.99, 1.00)**	0.99 (0.99, 0.99)**	1.00 (1.00, 1.00)*
Gender				
Female	ref	ref	ref	ref
Male	0.99 (0.97, 1.02)	0.91 (0.89, 0.93)**	1.20 (1.14, 1.26)**	0.96 (0.93, 1.00)*
Metropolitan residence				
Metropolitan	ref	ref	ref	ref
Non-metropolitan	1.01 (0.97, 1.04)	1.01 (0.97, 1.05)	1.03 (0.96, 1.11)	1.02 (0.97, 1.07)
Elixhauser comorbidity score				
0–1	ref	ref	ref	ref
2–3	1.23 (1.18, 1.28)**	1.11 (1.06, 1.16)**	0.96 (0.84, 1.09)	1.78 (1.68, 1.89)**
4 or more	2.77 (2.67, 2.87)**	2.14 (2.06, 2.23)**	1.14 (1.01, 1.27)	4.66 (4.41, 4.93)**

Significance levels: * < 0.05; ** < 0.001.

Table 4 shows results of the multinomial PSM analyses, estimating the average treatment effects on treated (ATET) for all four types of health care costs per patient based on previously receiving periodontal treatment when controlling for covariates (see Appendix C for binomial PSM analysis results). Reported percentage reductions and ATET dollar differences reflect estimates derived from PSM-weighted models. For those with four or more periodontal treatments, compared to those without any periodontal treatments, there was a 9.6% decrease in overall medical costs ($18,552 vs. $20,513; ATET = −$1960.39; 95% CI = −$2,684.33, −$1,236.44, *p* < 0.005), and a 5.3% decrease in outpatient costs ($10,733 vs. $11,332, ATET = −$599.09; 95% CI = −$1,019.34, −$178.84, *p* < 0.005). For those with one to three periodontal treatments, compared to those without any periodontal treatments, there was an 8% decrease in prescription costs ($4,429 vs. $4,816, ATET = −$387.53; 95% CI = −$653.31, −$119.75, *p* < 0.005). See Appendix D for results of the multinomial PSM analyses, estimating the ATET for all four types of health care costs per patient based on previously receiving periodontal treatment when controlling for covariates, across different CVD diagnoses. Only two findings across different CVD diagnoses emerged as statistically significant: individuals with cerebrovascular CVD who had 1–3 periodontal visits had higher overall medical costs (ATET = $3,727; 95% CI = $763, $6,690, *p* < 0.005) and individuals with arterial CVD who had 1–3 periodontal visits had lower overall medical costs ATET = −$2,330 (95% CI = −$4,311, −$348, *p* < 0.005).

**Table 4:** Multinomial propensity score matching analysis results for average overall medical, outpatient, inpatient, and prescription costs for patients with CVD with by number of periodontal treatments [average treatment effect (standard error); 95% confidence interval].

	Overall medical costs	Outpatient costs	Inpatient costs	Prescription costs
No periodontal visits	ref	ref	ref	ref
1–3 periodontal visits	−434.32 (368.98) *−1,157.50, 288.87*	−109.31 (214.21) *−529.15, 310.54*	2,545.30 (2,214) *−1,618.71, 6,709.34*	−386.53 (136.11)* −653.31, −119.75
4 or more periodontal visits	−1,960.39 (369.37)* −2,684.33, −1,236.44	−599.09 (214.42)* −1,019.34, −178.84	−27.3 (2,130.6) *−4,203.52, 4,148.93*	−79.06 (136.24) *−346.08,187.97*

Ref, Reference level; Significance levels: * < 0.005; model adjusted for age, gender, Elixhauser category, and metropolitan residence status.

As shown in Table 5, the multinomial PSM procedure resulted in substantial improvement in baseline covariate balance across periodontal treatment groups. All post-matching standardized mean differences were <0.10, with most reduced to 0.00–0.02, indicating excellent balance on age, gender, metropolitan residence, and Elixhauser comorbidity categories (Appendices E,F). These diagnostics support the validity of the matched sample and subsequent ATET estimates presented in Table 4.

**Table 5:** Standardized mean differences (SMD) for baseline covariates before and after multinomial propensity score matching for patients with CVD.

	SMD (Pre-Matching)	SMD (Post-Matching)
Age	0.27	0.02
Gender		
Female	ref	ref
Male	0.08	0.00
Metropolitan		
Metropolitan	ref	ref
Non-metropolitan	0.06	0.00
Elixhauser comorbidity score		
0–1	0.02	0.00
2–3	0.03	0.00
4+	0.40	0.00

ref, Reference level.

## Discussion

This study of patients with CVD found that patients with four or more periodontal visits in 2020–2021 had a 9.6% decrease in overall medical costs and a 5.3% decrease in outpatient costs in 2022 than those who did not have any periodontal visits. Compared to those without periodontal treatment, patients with one to three visits had an 8% decrease in prescription costs. These findings align with prior observational studies using similar designs (continuous enrollment with annual follow on periods), which demonstrate reduced inpatient and outpatient health care costs following at least one periodontal treatment for adults (18+) diagnosed with cerebrovascular disease (28), as well as decreased total, inpatient, outpatient, and drug costs for individuals with coronary heart disease who received at least one periodontal service (29).

The observed reduction in commercial health care costs associated with some form and frequency of periodontal treatment may reflect shared underlying inflammatory processes common to both conditions. Periodontal disease is a chronic inflammatory condition of which treatment, including non-surgical therapy, diet and lifestyle changes, and pharmaceutical drugs are intended to reduce such local inflammation (37). Because inflammation is also a feature of CVD progression, these overlapping biological processes have prompted ongoing research into possible shared pathways (17–21). Our observational findings are consistent with this context, though they do not establish causation. While evidence on the direct impact of periodontal treatment on CVD outcomes remains mixed (23, 24), our study adds to a growing body of research showing associations between periodontal care patterns and subsequent health care costs (indirect, inpatient, and outpatient) in adults with CVD (27, 28). These findings align with previous research indicating that non-surgical periodontal therapy has been associated with reductions in cardiovascular risk factors and inflammatory biomarkers (23, 24), reinforcing the potential value of periodontal care as part of broader strategies to control chronic disease and associated costs.

Most current studies that examine health care costs associated with periodontal treatment in adults with some form of CVD are based on whether participants received or did not receive at least one periodontal or preventive dental service (28–30). In contrast, our study analyzed health care costs for adults with some form of CVD who received periodontal treatment by more detailed frequency categories: none, 1–3, or 4 + periodontal services. A key finding of the current study is that a greater frequency of periodontal visits was associated with lower costs, though the direction of this association cannot be inferred. Specifically, individuals who received four or more periodontal treatments were observed to have significantly lower overall medical and outpatient costs, while those with one to three visits experienced notably reduced prescription costs compared to individuals who received no periodontal care.

These results reinforce the American Academy of Periodontology’s (AAP) recommendations that treatment for chronic periodontitis should include non-surgical therapy, including initial scaling and root planing and use of chemotherapeutic agents when indicated (38, 39). Furthermore, the findings support AAP guidelines and other studies suggesting that the frequency of ongoing dental recall visits, ranging from every 3 to 24 months, should be tailored to individual risk factors such as health behaviors, systemic conditions like CVD, and the management of periodontal–systemic relationships (40–42). Notably, a randomized controlled trial conducted in the United Kingdom found that a variable, risk-based recall interval was acceptable to both patients and providers, and showed no difference in clinical oral health outcomes, such as bleeding on probing, when compared to a fixed six-month recall schedule (43). Together, these findings highlight the potential relevance of personalized, risk-based periodontal care as a strategy for improving both health and cost outcomes. Increasing health care engagement through adherence to routine periodontal care can be complemented by strategies that are associated with lower health care costs, such as addressing shared cardiovascular and periodontal disease risk factors (e.g., smoking, poor diet and nutrition, overweight or obesity) (4, 12) and designing insurance plans that prioritize prevention.

Findings from this study suggest that periodontal treatment – particularly when delivered consistently – may be associated with lower observed overall medical, outpatient, and prescription costs among adults age 21–64 with commercial insurance diagnosed with CVD. Study results emphasize the importance of integrating oral health care, primary care, cardiology, and other health professions to improve whole-patient care and potentially reduce CVD-related costs (44–46). Prior work suggests providing oral health education to hospitalized CVD patients has been linked to improvements in patients’ oral health and CVD-related outcomes such as fewer days with post-operative atrial fibrillation (47), suggesting hospital staff should be trained in oral health education and/or employ oral health care providers (e.g., dental hygienists) in hospital settings. This study also lays the groundwork for expanding this type of analysis beyond CVD to other chronic conditions. There is a growing body of research that establishes a link between periodontal treatment and lower health care costs for individuals with diabetes (48–52). Findings from this study and others support further investigation into the associations between periodontal treatment and health care costs in other chronic conditions, such as rheumatoid arthritis and some forms of dementia (53). Finally, these study results and others suggest that dental insurance coverage for preventive care and periodontal treatment (particularly for at least 4 visits per year) could be cost-effective for the overall health care system in terms of reducing health care costs related to chronic conditions such as CVD and diabetes (54). Coverage policies among commercial plans that incentivize regular dental visits and periodontal maintenance may be linked to long-term differences in costs by reducing the burden of CVD-related complications and associated healthcare utilization. While this study did not review cost savings for those with Medicaid insurance, expanding adult Medicaid coverage for periodontal services should also be considered.

There were several limitations to this study. Over 80% of patients with CVD in this sample did not receive periodontal treatment. While there are no large-scale studies estimating how many adults in the United States *receive* periodontal treatment in a year, more than 40% of adults aged 30 and above have periodontitis (9, 10), twice the number of individuals in this sample who received periodontal treatment. This may limit the generalizability of conclusions drawn about patients receiving periodontal treatment in this sample. To have an adequate sample size, the analyses did not control for other comorbid conditions that may affect treatment costs for both periodontal disease and CVD, such as diabetes mellitus; future research should examine the association of health care costs and treatment for multiple comorbidities. This study observed that individuals who only had surgical periodontal treatment had higher overall medical and outpatient care costs than those with non-surgical or those with a mix of non-surgical and surgical periodontal treatment. The higher medical costs may be related to surgical periodontal interventions being generally more expensive than non-surgical treatment. Additionally, surgical periodontal treatment is typically indicated for those with more advanced diseased state where inflammation is more progressed and systemic. However, as dental diagnosis codes are not consistently available in claims data, it is not possible to determine the severity of patients’ periodontal disease. Though CVD is most common in older adults (ages 60+) (3) and the highest direct and indirect costs of CVD are among aged 80 and older (26), our analyses only included adults age 21–64 due to commercial claims data age limitations. Analyses controlled for age, gender, metropolitan residence, and Elixhauser comorbidities, but as other confounding variables such as socioeconomic status and access to dental care are not included in claims data, it was not possible to determine whether these factors played a role in the relationship between receiving periodontal treatment and health care costs. Because claims do not capture key behavioral and socioeconomic determinants such as education, income, smoking status, and oral hygiene behaviors, residual confounding remains possible even after propensity score matching and regression adjustment, particularly given the limited covariate set. These unmeasured factors, such as race/ethnicity, income, education, may influence both the likelihood of receiving periodontal care and subsequent health care costs, limiting the generalizability of the findings. Due to the observational nature of this analysis, causational conclusions cannot be drawn. Finally, the large sample size may yield statistically significant results, but it is not possible to determine whether the results are clinically significant.

### Future research directions

While evidence remains mixed regarding the role of periodontal treatment in the prevention and management of CVD, further research is needed to explore the causal pathways between periodontal care and CVD outcomes. Although there is support for regular, risk-based dental recall visits tailored to individual health and lifestyle factors, more longitudinal studies are necessary to compare the sustained impact of periodontal treatment versus prophylaxis recall visits in individuals without diagnosed periodontitis. Cost-effectiveness modeling would also help determine the balance of reduced health care costs within the context of increased costs of periodontal treatment.

Notably, in the current study, 80% of participants with CVD did not receive periodontal care. This finding suggests that the observed reductions in health care costs associated with periodontal treatment could be even more pronounced with broader access, especially given the high estimated prevalence of periodontitis across the U. S. population. Future research should prioritize strategies to expand access to and increase patient acceptance of periodontal care, while also exploring the potential cost savings that may result from greater utilization. Additionally, it is critical to investigate barriers to accessing periodontal treatment, particularly among high-risk populations including those with Medicaid insurance, who were not represented in this analysis. This will be especially critical for reducing the significant financial strain that CVD and periodontal disease place on the healthcare system.

## Conclusion

This study found that patients with CVD who had four or more periodontal visits showed a significant decrease in overall medical costs and outpatient costs compared to those who did not have any periodontal visits. Additionally, compared to those without periodontal treatment, patients with one to three periodontal visits showed a significant decrease in prescription costs. Although surgical-only periodontal treatment was not associated with reduced medical costs in this study, surgical treatment should be offered and provided as a treatment for periodontal disease when clinically appropriate, nonetheless. These findings emphasize the importance of better understanding the oral-systemic connection between CVD and periodontal disease as well as developing interventions to improve oral and overall health outcomes and reduce health care costs.

## Data Availability

The original contributions presented in the study are included in the article/Supplementary material, further inquiries can be directed to the corresponding author.
