# Supplementary material for: Periodontal therapy among patients with cardiovascular disease: exploring its association with lower medical spending
Source: Front Public Health. 2026 Mar 17;14:1741942. doi: 10.3389/fpubh.2026.1741942 (PMC13036156; doi:10.3389/fpubh.2026.1741942)
Supplement: Supplementary file 1 [file Data_sheet_1.docx]

**Appendix A:** ICD-10 codes within the I00-I99 range (Diseases of the Circulatory System)

| **Code** | **Description** |
| --- | --- |
| I00-I02 | Acute rheumatic fever |
| I05-I09 | Chronic rheumatic heart diseases |
| I10-I1A | Hypertensive diseases |
| I20-I25 | Ischemic heart diseases |
| I26-I28 | Pulmonary heart disease and diseases of pulmonary circulation |
| I30-I5A | Other forms of heart disease |
| I60-I69 | Cerebrovascular diseases |
| I70-I79 | Diseases of arteries, arterioles, and capillaries |
| I80-I89 | Diseases of veins, lymphatic vessels and lymph nodes, not elsewhere classified |
| I95-I99 | Other and unspecified disorders of the circulatory system |

**Appendix B:** Periodontal Treatment CDT Codes (D4000-4999) by Surgical vs. Non-Surgical Classification

**Surgical Services (Including Usual Postoperative Care)**

| Code | Description |
| --- | --- |
| D4210 | Gingivectomy or gingivoplasty – four or more contiguous teeth or tooth bounded spaces per quadrant |
| D4211 | Gingivectomy or gingivoplasty – one to three contiguous teeth or tooth bounded spaces per quadrant |
| D4212 | Gingivectomy or gingivoplasty to allow access for restorative procedure, per tooth |
| D4230 | Anatomical crown exposure – four or more contiguous teeth or tooth bounded spaces per quadrant |
| D4231 | Anatomical crown exposure – one to three teeth or tooth bounded spaces per quadrant |
| D4240 | Gingival flap procedure, including root planing - four or more contiguous teeth or tooth bounded spaces per quadrant |
| D4241 | Gingival flap procedure, including root planing - one to three contiguous teeth or tooth bounded spaces per quadrant |
| D4245 | Apically positioned flap |
| D4249 | Clinical crown lengthening – hard tissue |
| D4260 | Osseous surgery (including elevation of a full thickness flap and closure) – four or more contiguous teeth or tooth bounded spaces per quadrant |
| D4261 | Osseous surgery (including elevation of a full thickness flap and closure) – one to three contiguous teeth or tooth bounded spaces per quadrant |
| D4263 | Bone replacement graft – retained natural tooth – first site in quadrant |
| D4264 | Bone replacement graft – retained natural tooth – each additional site in quadrant |
| D4265 | Biologic materials to aid in soft and osseous tissue regeneration, per site |
| D4266 | Guided tissue regeneration, natural teeth – resorbable barrier, per site |
| D4267 | Guided tissue regeneration, natural teeth – non-resorbable barrier, per site |
| D4268 | Surgical revision procedure, per tooth |
| D4270 | Pedicle soft tissue graft procedure |
| D4273 | Autogenous connective tissue graft procedure (including donor and recipient surgical sites) first tooth, implant, or edentulous tooth position in graft |
| D4274 | Mesial/distal wedge procedure, single tooth (when not performed in conjunction with surgical procedures in the same anatomical area) |
| D4275 | Non-autogenous connective tissue graft (including recipient site and donor material) first tooth, implant, or edentulous tooth position in graft |
| D4276 | Combined connective tissue and pedicle graft, per tooth |
| D4277 | Free soft tissue graft procedure (including recipient and donor surgical sites) first tooth, implant or edentulous tooth position in graft |
| D4278 | Free soft tissue graft procedure (including recipient and donor surgical sites) each additional contiguous tooth, implant or edentulous tooth position in same graft site |
| D4283 | Autogenous connective tissue graft procedure (including donor and recipient surgical sites) – each additional contiguous tooth, implant or edentulous tooth position in same graft site |
| D4285 | Non-autogenous connective tissue graft procedure (including recipient surgical site and donor material) – each additional contiguous tooth, implant or edentulous tooth position in same graft site |
| D4286 | Removal of non-resorbable barrier |

**Non-Surgical Periodontal Services**

| D4322 | Splint – intra-coronal; natural teeth or prosthetic crowns |
| --- | --- |
| D4323 | Splint – extra-coronal; natural teeth or prosthetic crowns |
| D4341 | Periodontal scaling and root planing - four or more teeth per quadrant |
| D4342 | Periodontal scaling and root planing - one to three teeth per quadrant |
| D4346 | Scaling in presence of generalized moderate or severe gingival inflammation – full mouth, after oral evaluation |
| D4355 | Full mouth debridement to enable a comprehensive periodontal evaluation and diagnosis on a subsequent visit |
| D4381 | Localized delivery of antimicrobial agents via a controlled release vehicle into diseased crevicular tissue, per tooth |
| D4910 | Periodontal maintenance |
| D4920 | Unscheduled dressing change (by someone other than treating dentist or their staff) |
| D4921 | Gingival irrigation with a medicinal agent – per quadrant |
| D4999 | Unspecified periodontal procedure, by report |

**Appendix C.** Binomial Propensity Score Matching Analysis Results for Average Overall Medical, Outpatient, Inpatient, and Prescription Costs for Patients with CVD by Having Had at Least One or More Periodontal Visits (Average Treatment Effect (Standard Error); *95% Confidence Interval*)

|  | Overall Medical  Costs | Outpatient Costs | Inpatient Costs | Prescription Costs |
| --- | --- | --- | --- | --- |
| No periodontal visit | *ref* | *ref* | *ref* | *ref* |
| One or more periodontal visits | -1,103.1  (494.50)*  *-2,072.3, -133.9* | -226.80  (280.40)  *-776.5, 322.9* | -602.70  (295.40)*  *-1,181.7, -23.7* | -273.60 (186.20)  *-638.5, 91.3* |

*ref* = reference level; significance levels = *<0.005; model adjusted for age, gender, Elixhauser category, and metropolitan residence status.

**Appendix D.** Multinomial Propensity Score Matching Analysis Results on Average Treatment Effect on Treated for Overall Medical Costs for Patients with CVD with by Number of Periodontal Treatments and CVD Type (Average Treatment Effect (Standard Error); *95% Confidence Interval*)

|  | Hypertensive CVD | Ischemic CVD | | Pulmonary CVD | | Cerebrovascular CVD |
| --- | --- | --- | --- | --- | --- | --- |
| No periodontal visits | *ref* | *ref* | | *ref* | | *ref* |
| 1-3 periodontal visits | -1,015.20 (1,370.40)  *-3,701.35, 1,670.97* | -325.34 (634.90)  *-1,569.74,919.10* | | -4,009.90 (2,724.70)  *-9,352.25,1,332.36* | | 3,727.01 (1,511.88)*  *763.37,6,690.66* |
| 4 or more periodontal visits | -1,571.80 (1,373.40)  *-4,264.88, 1,119.27* | -178 (634.92)  *-1,422.46,1,066.49* | | -4,201.90 (2,780.60)  *-9,653.81,1,250.09* | | -2,311.65 (1,519.79)  *-5,290.79,667.49* |
|  | Arterial CVD | | Venous CVD | | Other CVD | |
| No periodontal visits | *ref* | | *ref* | | *ref* | |
| 1-3 periodontal visits | -2,330 (1,010.99)  *-4,311,67, -348.33* | | -1,742.64 (2,749.35)  *-7,134.89, 3,649.61* | | 820.92 (597.89)  *-350.95, 1,992.79* | |
| 4 or more periodontal visits | -716.84 (1,012.33)  -2,701.15, 1,267.46 | | -4,129.65 (2,741.61)  *-9,506.72, 1,247.41* | | -986.76 (599.06)  *-2,160.93, 187.41* | |

*ref* = reference level; significance levels = *<0.005; model adjusted for age, gender, Elixhauser category, and metropolitan residence status.

**Appendix E**. Standardized Mean Differences (SMDs) for Covariates Before and After Binary Propensity Score Matching

|  | SMD (Pre-Matching) | SMD (Post-Matching) |
| --- | --- | --- |
| Age | 0.15 | 0.00 |
| Gender |  |  |
| Female | *ref* | *ref* |
| Male | 0.05 | 0.00 |
| Metropolitan |  |  |
| Metropolitan | *ref* | *ref* |
| Non-metropolitan | 0.06 | 0.00 |
| Elixhauser Comorbidity Score |  |  |
| 0-1 | -0.02 | 0.00 |
| 2-3 | -0.02 | 0.00 |
| 4+ | -0.01 | 0.00 |

*ref* = reference level

**Appendix F.** Standardized Mean Differences for Baseline Covariates Before and After Propensity Score Matching, Stratified by Cardiovascular Disease Subtype

|  | Hypertensive CVD | | Ischemic CVD | | Pulmonary CVD | |
| --- | --- | --- | --- | --- | --- | --- |
|  | SMD (Pre-Matching) | SMD (Post-Matching) | SMD (Pre-Matching) | SMD (Post-Matching) | SMD (Pre-Matching) | SMD (Post-Matching) |
| Age | 0.27 | 0.04 | 0.23 | 0.03 | 0.27 | 0.09 |
| Gender |  |  |  |  |  |  |
| Female | *ref* | *ref* | *ref* | *ref* | *ref* | *ref* |
| Male | 0.05 | 0.00 | 0.06 | 0.00 | 0.08 | 0.01 |
| Metropolitan |  |  |  |  |  |  |
| Metropolitan | *ref* | *ref* | *ref* | *ref* | *ref* | *ref* |
| Non-metropolitan | 0.07 | 0.01 | 0.07 | 0.01 | 0.08 | 0.05 |
| Elixhauser Comorbidity Score |  |  |  |  |  |  |
| 0-1 | 0.00 | 0.00 | 0.02 | 0.00 | 0.04 | 0.01 |
| 2-3 | 0.02 | 0.00 | 0.01 | 0.00 | 0.02 | 0.00 |
| 4+ | 0.02 | 0.01 | 0.01 | 0.00 | 0.03 | 0.01 |
|  | Arterial CVD | | Venous CVD | | Other CVD | |
|  | SMD (Pre-Matching) | SMD (Post-Matching) | SMD (Pre-Matching) | SMD (Post-Matching) | SMD (Pre-Matching) | SMD (Post-Matching) |
| Age | 0.31 | 0.03 | 0.34 | 0.05 | 0.34 | 0.05 |
| Gender |  |  |  |  |  |  |
| Female | *ref* | *ref* | *ref* | *ref* | *ref* | *ref* |
| Male | 0.08 | 0.00 | 0.13 | 0.04 | 0.13 | 0.04 |
| Metropolitan |  |  |  |  |  |  |
| Metropolitan | *ref* | *ref* | *ref* | *ref* | *ref* | *ref* |
| Non-metropolitan | 0.06 | 0.01 | 0.07 | 0.03 | 0.07 | 0.03 |
| Elixhauser Comorbidity Score |  |  |  |  |  |  |
| 0-1 | 0.03 | 0.00 | 0.05 | 0.01 | 0.05 | 0.01 |
| 2-3 | 0.05 | 0.00 | 0.06 | 0.01 | 0.06 | 0.01 |
| 4+ | 0.07 | 0.00 | 0.01 | 0.02 | 0.01 | 0.02 |

*ref* = reference level
